# The global immune-nutrition-inflammation index predicts pathological response and survival in esophageal squamous cell carcinoma treated with neoadjuvant immunochemotherapy

**DOI:** 10.3389/fnut.2025.1717477

**Published:** 2025-12-03

**Authors:** Zhouxv Feng, Huihan Yi, Jiazhou Xiao, Zhixin Huang, Yifei Tu, Bo Liu

**Affiliations:** 1 Department of Thoracic Surgery, The First Affiliated Hospital, Fujian Medical University, Fuzhou, China; 2 Department of Thoracic Surgery, National Regional Medical Center, Binhai Campus of the First Affiliated Hospital, Fujian Medical University, Fuzhou, China

**Keywords:** global immune-nutrition-inflammation index (GINI), esophageal squamous cell carcinoma, neoadjuvant immunochemotherapy, prognosis, biomarker

## Abstract

**Objective:**

The global immune-nutrition-inflammation index (GINI) is a new composite indicator that assesses nutrition and inflammation and has been linked to prognosis in various cancers. Neoadjuvant immunochemotherapy (nICT) is becoming more common for treating locally advanced esophageal squamous cell carcinoma (ESCC). However, the potential of GINI to predict outcomes for ESCC patients undergoing nICT has not yet been explored. This study aims to examine the predictive value of the pretreatment GINI in relation to pathological response and prognosis for ESCC patients receiving nICT.

**Methods:**

A total of 138 patients with locally advanced ESCC who underwent nICT followed by radical resection at our institution between 2022 and 2024 were retrospectively included in this study. The GINI index was calculated from pretreatment blood parameters. The receiver operating characteristic (ROC) curve analysis was conducted to determine the optimal cutoff value of the GINI index for predicting pathological response, which was defined using the Becker tumor regression grade (TRG). Logistic regression and Cox proportional hazards models were used to analyze the associations between the GINI index and both pathological response and survival outcomes.

**Results:**

The optimal cutoff value of GINI for predicting pathological response was 73.47 (AUC = 0.912). Multivariate analysis identified high-GINI as an independent risk factor for both poor pathological response (OR = 1.05, *p* < 0.001) and shorter overall survival (OS) (HR = 1.01, *p* = 0.012). Compared to the low-GINI group, patients in the high-GINI group had significantly poorer tumor differentiation, more advanced pathological stage, and a higher incidence of complications (all *p* < 0.05). Survival analysis demonstrated that the low-GINI group had significantly better 3-year OS (87.8% vs. 68.7%, *p* = 0.014) and disease-free survival (DFS) (82.7% vs. 63.3%, *p* = 0.011) than the high-GINI group.

**Conclusion:**

Pretreatment GINI is a promising biomarker for predicting pathological response and survival outcomes in locally advanced ESCC patients treated with nICT. A high GINI level is significantly associated with treatment resistance and poorer prognosis, suggesting its potential utility in risk stratification and guiding individualized treatment strategies.

## 1 Introduction

Esophageal cancer (EC) is a significant global public health challenge. In 2022, there were approximately 511,000 new cases and 445,000 deaths worldwide, placing its incidence and mortality as the 11th and 7th highest, respectively, among all malignancies globally (1). While the incidence of esophageal cancer in China has been declining due to early screening and better control of risk factors, the country still bears one of the highest burdens of Esophageal Squamous Cell Carcinoma (ESCC) worldwide. The anatomical structure of the esophagus, combined with the non-specific early symptoms—such as difficulty swallowing (dysphagia) and chest pain (retrosternal pain)—often leads to most patients being diagnosed at an advanced or metastatic stage (2). This late diagnosis significantly affects treatment options and patient prognosis. In recent years, neoadjuvant immunochemotherapy (nICT) has emerged as a crucial treatment strategy for locally advanced ESCC. This approach can downstage tumors, eliminate micrometastases, and reduce the risks of intraoperative dissemination and postoperative recurrence, potentially improving patient outcomes. However, due to the heterogeneity of tumors, not all patients benefit from nICT. Current data suggest that only about 20 to 40% of patients achieve an optimal pathological response, with an even smaller percentage attaining long-term disease control (3). Therefore, it is essential to identify effective biomarkers that can predict both the pathological response and prognosis of nICT.

In clinical management, patients with EC face significant nutritional management challenges, as dysphagia often leads to a high incidence of malnutrition and cachexia. Concurrently, side effects from essential treatments such as chemotherapy, radiotherapy, and surgery—including anorexia and metabolic consumption—synergize with disease-related factors, further exacerbating the decline in nutritional status. As a result, the relationship between nutritional status and cancer prognosis has garnered increasing attention from researchers (4). To assess and predict prognosis in esophageal cancer, several nutritional assessment tools have been utilized, including the Prognostic Nutritional Index (PNI) (5), the global immune-nutrition-inflammation index (GINI) (6), Geriatric Nutritional Risk Index (GNRI) (7), Controlling Nutritional Status (CONUT) score (8), and Nutritional Risk Screening 2002 (NRS2002) score (9).

GINI is a composite indicator that combines six hematological parameters: C-reactive protein, platelets, monocytes, neutrophils, albumin, and lymphocytes. This index allows for a comprehensive assessment of the body’s nutritional and inflammatory status. It was initially developed and validated by Topkan et al. (10) as a prognostic factor for stage IIIC non-small cell lung cancer (NSCLC) patients undergoing chemoradiotherapy. Subsequently, its predictive value has been further confirmed in various gastrointestinal malignancies, including gastric cancer (11), esophageal cancer (6), and colorectal cancer (12), demonstrating a close association with tumor progression and outcomes.

Currently, no studies have examined the clinical value of GINI in patients with ESCC who are receiving nICT. To fill this research gap, this study is the first to systematically investigate the predictive role of GINI concerning treatment effectiveness and prognosis in ESCC patients undergoing nICT. The aim is to clarify its potential as a novel biomarker.

## 2 Materials and methods

### 2.1 Participants

We conducted a retrospective analysis of patients with locally advanced ESCC who received nICT at our institution from January 2022 to April 2024. The inclusion criteria were as follows: (1) age 18–75 years; (2) pathologically confirmed ESCC; (3) an Eastern Cooperative Oncology Group performance status (ECOG-PS) score of 0–1; (4) clinical TNM stage II-IVA; (5) successful R0 radical resection following nICT; and (6) availability of complete clinical data with a follow-up duration of ≥6 months. The exclusion criteria were: (1) pathological type other than ESCC; (2) failure to achieve R0 resection after nICT (i.e., R1 or R2 resection); (3) concomitant active infection, hematological disease, or autoimmune disease; (4) history of or concurrent other primary malignant tumors; or (5) receipt of other antitumor therapies during the study period. The study protocol was approved by the Medical Ethics Committee of the First Affiliated Hospital of Fujian Medical University (Approval No.: [2015]084–2) and strictly adhered to the ethical principles of the Declaration of Helsinki (2013 revision). Since this was a retrospective analysis, all patient data were anonymized, and retrospective informed consent for data usage was obtained, ensuring compliance with patient privacy and ethical standards.

**Figure 1:**
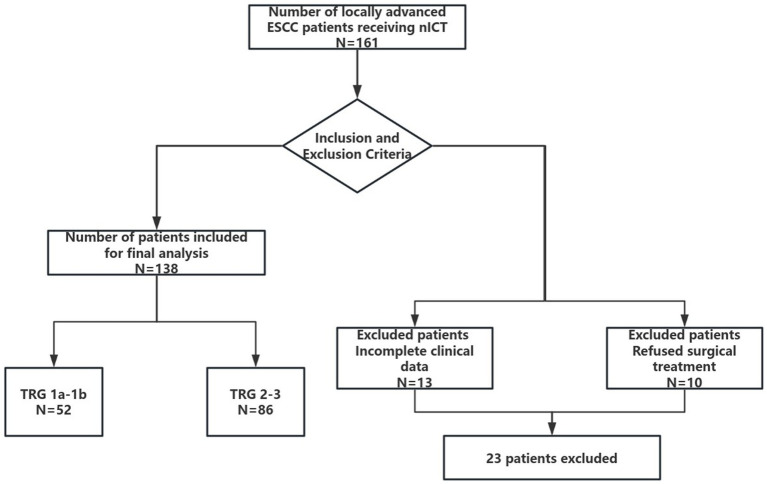
Flow chart showing patient recruitment.

A total of 161 patients with ESCC underwent nICT. Among them, 10 patients refused further treatment, and 13 patients had incomplete clinical data. Therefore, this study retrospectively included 138 patients (Figure 1).

### 2.2 Treatment protocols

All patients received nICT according to the Chinese Society of Clinical Oncology (CSCO) guidelines for esophageal cancer. The regimen consisted of albumin-bound paclitaxel (135 mg/m^2^, day 1), cisplatin (75 mg/m^2^, day 1), and tislelizumab/sintilimab/camrelizumab (200 mg, day 2), administered every 3 weeks for two cycles. Surgical resection was performed 4–6 weeks after the last nICT cycle. The surgical approach was determined by a multidisciplinary team (MDT) discussion and included minimally invasive esophagectomy (MIE, incorporating thoracoscopic/laparoscopic approaches), open surgery, or robot-assisted procedures, selected based on individual patient characteristics. All surgeries were performed by an experienced thoracic surgery team (averaging over 100 esophagectomies annually) at our institution.

### 2.3 Follow-up

To monitor disease progression, all patients were enrolled in a regular follow-up program after completion of treatment: follow-ups were conducted every 3 months for the first 2 years postoperatively, every 6 months during years 3–5, and annually thereafter. This study utilized the 8th edition AJCC/UICC staging system, and data collection continued until June 2025.

### 2.4 Calculation of the GINI

GINI is a composite parameter calculated based on six hematological indicators: neutrophil count (NEU#), monocyte count (MON#), platelet count (Plt), lymphocyte count (LYM#), albumin level, and C-reactive protein (CRP) level. In accordance with established research protocols, peripheral blood was collected from patients within 1 week prior to initiating nICT, and data for the aforementioned parameters were extracted. The GINI is calculated using the following formula:


$$\text{GINI}=\frac{\mathrm{CRP}(\mathrm{mg}/L)\times \mathrm{MON}\#(10^{9}/L)\times \mathrm{NEU}\#(10^{9}/L)\times \mathrm{Plt}(10^{9}/L)}{\text{Albumin}(g/L)\times \mathrm{LYM}\#(10^{9}/L)}.$$

### 2.5 Assessment of treatment response

Postoperative tumor regression was graded using the Becker Tumor Regression Grading (13), with the following criteria: TRG Grade 1a (Complete Regression): No residual tumor cells; TRG Grade 1b (Near-Complete Regression): Residual tumor cells occupy <10% of the tumor bed area; TRG Grade 2 (partial regression): residual tumor cells occupy 10–50% of the tumor bed area; TRG Grade 3 (poor regression): residual tumor cells occupy >50% of the tumor bed area. Pathological response to therapy was stratified as favorable (TRG 1a–1b, representing significant tumor remission) or suboptimal (TRG 2–3). For statistical analysis, patients were stratified by tumor regression grade (TRG) into two groups: TRG 1a–1b and TRG 2–3.

### 2.6 Statistical analysis

All cases included in this study underwent rigorous screening to ensure the completeness of key variables, including hematologic parameters and clinical pathological variables used for GINI calculation. Consequently, no missing values existed in the final analysis dataset, eliminating the need for imputation or exclusion.

Statistical analyses were performed using IBM SPSS Statistics software (Version 26.0) and R (version 4.x). Continuous data conforming to a normal distribution are presented as mean ± standard deviation and were compared using the Student’s *t*-test. Categorical data are presented as numbers (percentages) and were compared using the Chi-square test or Fisher’s exact test (when expected frequencies were <5). To evaluate the predictive ability of GINI for treatment response, a receiver operating characteristic (ROC) curve was plotted, and the area under the curve (AUC) was calculated in R. To assess further the model’s internal validation performance and optimism bias, 1,000 bootstrap resamples were performed, and corrected performance metrics including accuracy, Kappa value, sensitivity, specificity, and AUC were computed. The optimal cutoff value for GINI was determined using the Youden’s index. Variables showing statistical significance in univariate analysis were subsequently included in a multivariate logistic regression model to identify independent predictors of pathological response. The Cox proportional hazards regression model was used to analyze independent prognostic factors for disease-free survival (DFS) and overall survival (OS), with results expressed as hazard ratios (HRs) and 95% confidence intervals (CIs). Survival curves were plotted using the Kaplan–Meier method, the median follow-up time was calculated using the reverse Kaplan–Meier method, and intergroup comparisons were performed using the log-rank test. To enhance the clinical interpretability of the GINI index’s effect size, we standardized it using different units to recalculate the effect sizes: per standard deviation change (61.30 units), per 25-unit change, and per 50-unit change. Additionally, we assessed the linear relationship between the GINI index and outcome measures using the chi-square linear-by-linear association test and the Log-rank test for trend. All tests were two-sided, and a *p*-value < 0.05 was considered statistically significant.

## 3 Results

### 3.1 Patient characteristics

**Figure 2:**
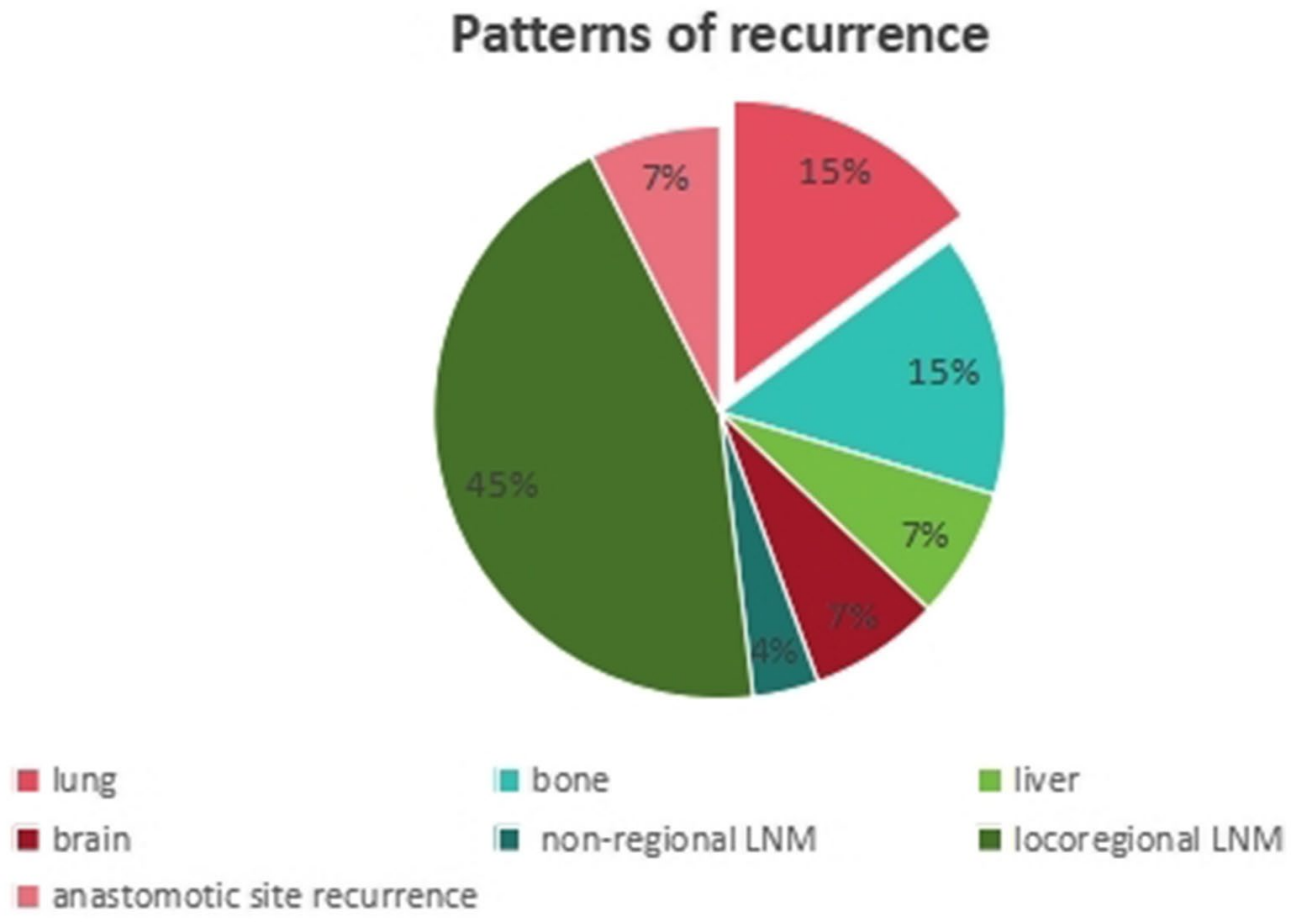
Detailed recurrence patterns after nICT. There were 13 (48.1%) cases with distant recurrence and 14 (51.9%) cases with local recurrence.

This study ultimately enrolled 138 patients with locally advanced ESCC who received nICT. The mean age of the entire cohort was 60.5 years (range: 47–75 years), comprising 121 males (87.7%) and 17 females (12.3%). The median follow-up time was 24.5 months (range: 6–41 months). Tumor locations were predominantly in the middle esophagus (50.7%) and lower esophagus (36.2%). The majority of tumors (58.0%) were moderately differentiated, while 16.7% were poorly differentiated. Postoperative pathological (ypT) staging was as follows: ypT0 in 26 cases (18.8%), ypT1-2 in 56 cases (40.6%), and ypT3-4a in 56 cases (40.6%). Postoperative complications occurred in 55 patients (39.9%). During the follow-up period, 27 (19.6%) patients experienced recurrence. Among them, there were 13 (48.1%) patients with distant recurrence after treatment, including peritoneal metastasis and non-regional lymph node metastasis (LNM), while there were 14 (51.9%) cases with local recurrence, including locoregional LNM and anastomotic site recurrence (Figure 2). Patient clinicopathological characteristics are shown in Table 1.

**Table 1:** Clinicopathological characteristics of 138 patients with esophageal squamous cell carcinoma.

Characteristics	Value
Gender, *n* (%)	
Male	121 (87.7)
Female	17 (12.3)
Mean age [range], years	60.5 (42, 75)
BMI mean [range], kg/m^2^	22.4 (16.2–30.9)
Hypertension history, *n* (%)	52 (37.6)
Diabetes history, *n* (%)	16 (11.5)
Smoking history, *n* (%)	56 (40.5)
Drinking history, *n* (%)	53 (38.4)
ECOG, *n* (%)	
0	117 (84.8)
1	21 (15.2)
Tumor length [range], cm	6.88 (2.6, 13.5)
Tumor location, *n* (%)	
Upper	18 (13.1)
Middle	70 (50.7)
Lower	50 (36.2)
Differentiation, *n* (%)	
Well	35 (25.3)
Moderate	80 (58.0)
Poor	23 (16.7)
Vessel invasion, *n* (%)	24 (17.4)
Perineural invasion, *n* (%)	38 (27.5)
Mean time to surgery [range], min	341.67 (180–485)
Mean blood [range], mL	151.23 (50–400)
Postoperative complications, *n* (%)	55 (39.9)
Pneumonia	34 (24.6)
Pleural effusion	26 (18.8)
Chylothorax	0 (0)
Cardiac events	13 (9.4)
Palsy of recurrent laryngeal nerve	0 (0)
Anastomotic leakage	10 (7.2)
Anastomotic narrow	0 (0)
Bleeding	3 (2.2)
Clinical TNM stage (per inclusion criteria), *n* (%)	
I	0 (0)
II	33 (23.9)
III	90 (65.2)
IV	15 (10.9)
ypT stage, *n* (%)	
T0	26 (18.8)
T1-2	56 (40.6)
T3-4a	56 (40.6)
ypN stage, *n* (%)	
N0	85 (61.6)
N1	35 (25.4)
N2-3	18 (13.0)
Tumor regression grade, *n* (%)	
TRG 1a	25 (18.1)
TRG 1b	27 (19.6)
TRG 2	25 (18.1)
TRG 3	61 (44.2)
Mean Albumin, g/L, [range]	42.4 (32.6–51.3)
Mean CRP, mg/L, [range]	21.32 (1.08–230.39)
Mean Monocyte count, 10^9^,[range]	0.43 (0.11–0.87)
Mean Platelet count, 10^9^, [range]	254.77 (77–434)
Mean Neutrophil count, 10^9^, [range]	4.43 (1.52–14.64)
Mean Lymphocyte count, 10^9^, [range]	1.61 (0.58–3.08)
Mean GINI, [range]	94.2 (8.12–307.73)

### 3.2 Univariate and multivariate analyses of pathological tumor regression response

To identify predictors of pathological tumor regression response, univariate and multivariate logistic regression analyses were performed (results shown in Table 2). Univariate analysis demonstrated that age (*p* = 0.025), lymphocyte count (LYM#, *p* = 0.021), and GINI score (*p* < 0.001) were significantly associated with pathological response. Multivariate analysis further confirmed that both LYM# (OR = 0.29, 95% CI: 0.09–0.90, *p* = 0.033) and GINI score (OR = 1.05 95% CI: 1.03–1.06, *p* < 0.001) were independent predictive factors for pathological tumor regression following nICT.

**Table 2:** Logistic regression analysis of predictors for nICT efficacy.

Variables	Univariate analysis		Multivariate analysis	
	*p*	OR (95% CI)	*p*	OR (95% CI)
Age (years, >65 vs. ≤65)	0.025	0.43 (0.21–0.90)	0.117	0.94 (0.88–1.02)
Sex (male vs. female)	0.828	1.12 (0.39–3.25)		
BMI (Kg/m^2^, >20 vs. ≤20)	0.833	0.99 (0.88–1.11)		
Tumor location	0.284	1.33 (0.79–2.24)		
ECOG-PS (1 vs. 0)	0.966	1.02 (0.39–2.66)		
Smoking history (yes vs. no)	0.301	1.45 (0.72–2.91)		
Drinking history (yes vs. no)	0.477	0.77 (0.38–1.58)		
Hypertension history (yes vs. no)	0.194	0.62 (0.30–1.28)		
Diabetes history (yes vs. no)	0.109	0.34 (0.09–1.27)		
Albumin (g/L)	0.811	1.01 (0.91–1.12)		
NEU# (10^9^/L)	0.208	0.88 (0.73–1.07)		
LYM# (10^9^/L)	0.021	0.43 (0.21–0.88)	0.033	0.29 (0.09–0.90)
MON# (10^9^/L)	0.419	0.37 (0.32–4.17)		
Plt (10^9^/L)	0.557	1.00 (0.99–1.00)		
CRP (mg/L)	0.067	0.99 (0.97–1.00)		
GINI	<0.001	1.04 (1.03–1.06)	<0.001	1.05 (1.03–1.06)

### 3.3 Optimal cut-off values of GINI before nICT

**Figure 3:**
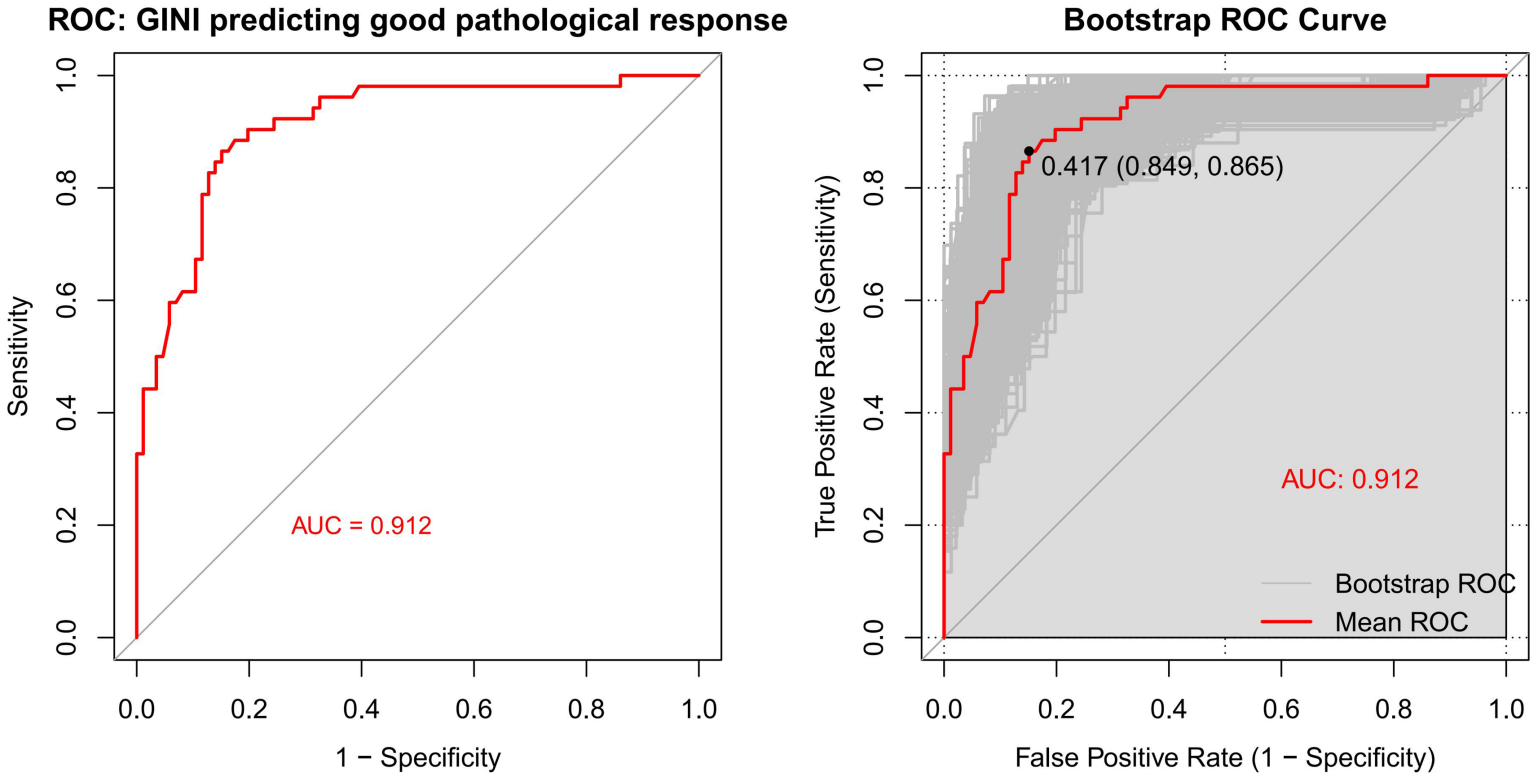
The left figure displays a single ROC curve based on the entire sample, while the right figure shows the results of 1,000 Bootstrap validations. The gray lines represent the ROC curve for each resampling, and the red line indicates the averaged ROC curve along with its corresponding AUC value. GINI, the global immune-nutrition-inflammation index; ROC, receiver operating characteristic.

To evaluate the predictive efficacy of GINI for tumor regression response, we constructed a ROC curve (Figure 3). The results demonstrated significant predictive value for GINI, with an area under the curve (AUC) of 91.2%. The optimal cutoff value was determined to be 73.47, corresponding to a sensitivity of 86.5%, specificity of 84.9%, and a Youden’s index of 0.714. And bootstrap internal validation (1,000 repetitions) demonstrated robust discriminative ability of the model, with a corrected mean AUC of 0.909, which is closely aligned with the original AUC of 0.912. Furthermore, the model exhibited strong predictive accuracy (accuracy = 0.840, Kappa = 0.658) and stable classification performance (sensitivity = 86.4%, specificity = 80.3%), indicating excellent generalizability and a low risk of overfitting. To assess the robustness of the identified optimal cutoff value (73.47), we performed a sensitivity analysis by evaluating the performance of adjacent cutoff values. As summarized in Supplementary Table 2, the predictive performance remained highly stable across a range of cutoff values from 72.72 to 75.42. Specifically, sensitivity ranged from 84.6 to 88.5%, specificity from 82.6 to 84.9%, and the negative predictive value consistently exceeded 90% at all tested thresholds. This stability confirms that the discriminative ability of the GINI index is not critically dependent on a single cutoff value and reinforces its reliability for clinical risk stratification.

Patients were dichotomized based on this cutoff into low-GINI (<73.47; *n* = 58) and high-GINI (≥73.47; *n* = 80) groups. Comparison of therapeutic efficacy between groups revealed that 77.6% of patients in the low-GINI group achieved a favorable pathological response, compared to only 8.6% in the high-GINI group. This difference was statistically significant (*p* < 0.001), indicating that a higher baseline GINI level is significantly associated with poorer nICT efficacy.

To enhance the clinical interpretability of the GINI index, we further analyzed its effect on outcomes using a continuous scale with standardized and clinically relevant units (Supplementary Table 1). In multivariate analyses, each one-standard-deviation increase in GINI (61.30 units) was associated with a significantly increased risk of poor pathological response (OR = 15.252, 95% CI: 6.216–37.422, *p* < 0.001) and worse overall survival (HR = 1.909, 95% CI: 1.155–3.156, *p* = 0.012). When scaled to smaller, clinically practical increments, each 25-unit and 50-unit increase in GINI remained significantly associated with adverse outcomes for both pathological response and survival (all *p* < 0.05).

Furthermore, tests for linear trend confirmed a significant dose–response relationship between the continuous GINI index and both pathological response (linear-by-linear association *χ*^2^ = 64.122, *p* < 0.001) and overall survival (log-rank test for trend *χ*^2^ = 7.147, *p* = 0.008), supporting its use as a robust and monotonically increasing risk factor.

### 3.4 Patient characteristics stratified by GINI

Based on the optimal cutoff value (73.47) determined by the ROC curve, the 138 patients were divided into a low-GINI group (58 patients, 42.0%) and a high-GINI group (80 patients, 58.0%). A comparison of the baseline clinical characteristics between the two groups is shown in Table 3. The high-GINI group showed significantly higher proportions of patients with poor tumor differentiation (*p* < 0.001), vascular invasion (*p* < 0.001), perineural invasion (*p* < 0.001), postoperative pneumonia (*p* = 0.016), pleural effusion (*p* = 0.014), as well as more advanced ypT stage (*p* < 0.001) and ypTNM stage (*p* < 0.001) compared to the low-GINI group.

**Table 3:** Comparison of clinical variables in esophageal squamous cell carcinoma patients stratified by GINI.

Characteristics	Low GINI (*N* = 58)	High GINI (*N* = 80)	*p*-value
Age (mean, years)	62.74 ± 7.40	59.18 ± 8.43	0.811
Sex (female/male)	8/50	9/71	0.794
ECOG-PS (0/1)	49/9	68/12	0.933
Tumor location (U/M/L)	10/29/19	8/41/31	0.428
Differentiation (W/M/P)	25/20/13	10/60/10	<0.001
Vessel invasion (no/yes)	57/1	57/23	<0.001
Perineural invasion (no/yes)	51/7	49/31	<0.001
Postoperative complications (no/yes)	43/15	39/41	0.003
Anastomotic leak (no/yes)	56/2	72/8	0.192
Pleural effusion (no/yes)	53/5	59/21	0.014
Cardiac events (no/yes)	52/6	73/7	0.775
Pneumonia (no/yes)	50/8	54/26	0.016
ypT stage (T0/T1-2/T3-4a)	24/25/9	2/31/47	<0.001
ypN stage (N0/N1/N2-3)	49/9/7	43/26/11	0.055
ypTNM stage (0/I-II/III-Iva)	24/21/13	2/44/34	<0.001

### 3.5 Survival analysis

**Figure 4:**
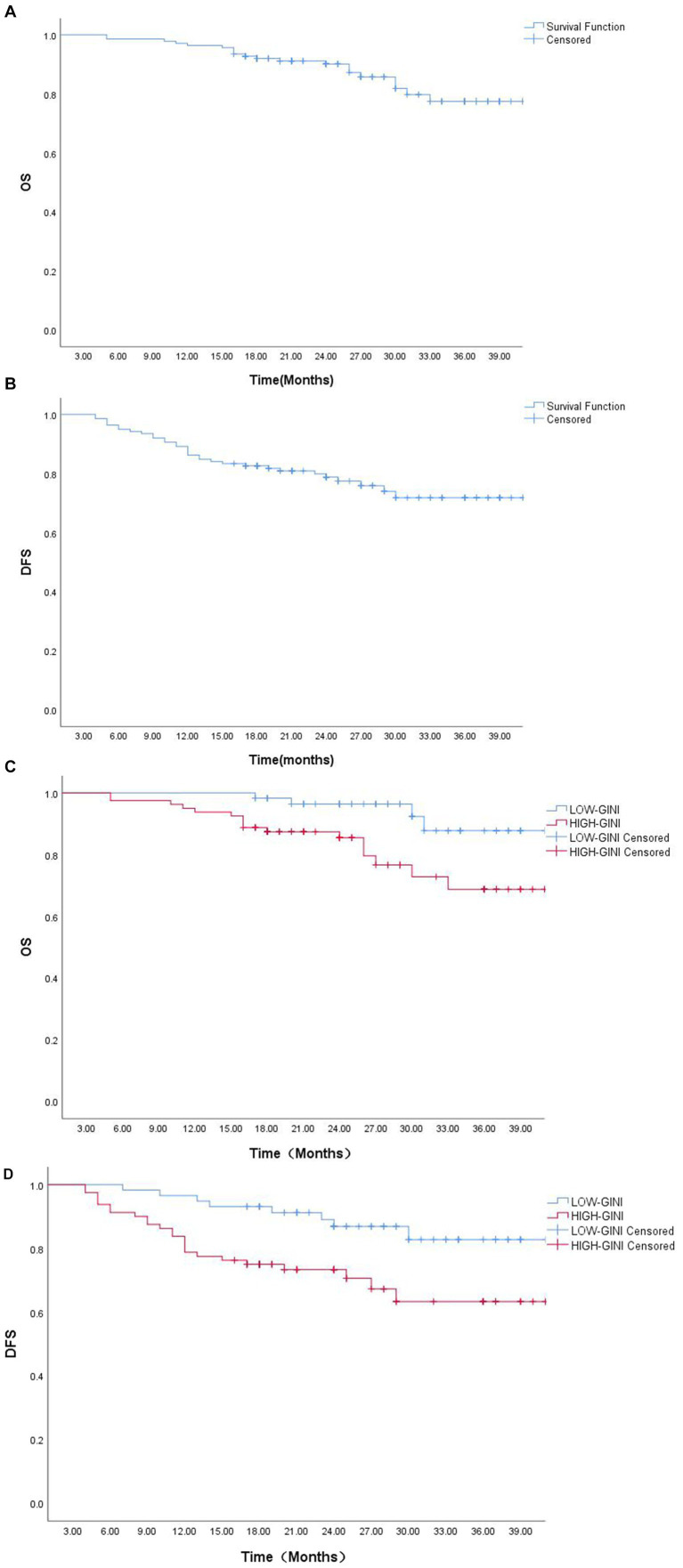
Survival analysis curves: **(A)** Overall survival (OS) curve for the entire cohort; **(B)** Disease-free survival (DFS) curve for the entire cohort; **(C)** OS curve stratified by GINI; **(D)** DFS curve stratified by GINI.

At the time of data analysis, the median survival time of this study had not been reached. For the entire cohort, the 1-, 2-, and 3-year survival rates were as follows: overall survival (OS), 96.4, 90.1, and 77.5%; disease-free survival (DFS), 86.2, 78.8, and 71.8%. Kaplan–Meier survival analysis (Figure 4) demonstrated that the low-GINI group had significantly better 3-year OS (87.8% vs. 68.7%, *p* = 0.014) and DFS (82.7% vs. 63.3%, *p* = 0.011) compared to the high-GINI group, indicating that a higher pretreatment GINI is a predictor of poor survival outcomes.

To further investigate the predictive value of GINI for survival outcomes following nICT, we conducted separate Cox regression analyses for DFS and OS. Univariate analysis for DFS initially identified six potential prognostic factors (Table 4). After adjusting for potential confounders, multivariate analysis identified only lymph node metastasis status, ypTNM stage, and postoperative complications as independent predictors of DFS. In contrast, for OS, multivariate analysis demonstrated that the GINI score (HR = 1.01, *p* = 0.012) exhibited independent prognostic value, highlighting its significant role in predicting long-term patient survival.

**Table 4:** Univariate and multivariate cox proportional hazards analyses of clinicopathological factors for DFS and OS.

Variables	DFS				OS			
	Univariate		Multivariate		Univariate		Multivariate	
	HR (95% CI)	*p*-value	HR (95% CI)	*p*-value	HR (95% CI)	*p*-value	HR (95% CI)	*p*-value
Age (years, >65 vs. ≤65)	1.63 (0.73–3.63)	0.234			1.09 (0.43–2.72)	0.860		
Sex (male vs. female)	0.68 (0.21–2.24)	0.526			1.13 (0.33–3.88)	0.843		
ECOG (1 vs. 0)	1.34 (0.55–3.25)	0.523			1.90 (0.69–5.23)	0.214		
Smoking history (yes vs. no)	1.64 (0.82–3.28)	0.165			1.30 (0.54–3.13)	0.557		
Drinking history (yes vs. no)	1.72 (0.86–3.44)	0.126			1.70 (0.71–4.11)	0.232		
Hypertension history (yes vs. no)	1.51 (0.70–3.26)	0.298			1.75 (0.64–4.82)	0.279		
Diabetes history (yes vs. no)	1.30 (0.39–4.25)	0.670			1.10 (0.26–4.75)	0.897		
T stage (T0 + T1 + T2 vs. T3-T4)	3.26 (1.57–6.77)	0.002	1.32 (0.58–3.02)	0.509	4.18 (1.60–10.91)	0.003	1.83 (0.64–5.20)	0.257
N stage (N0 + N1 vs. N2 + N3)	6.35 (3.12–12.91)	<0.001	3.39 (1.41–8.12)	0.006	4.61 (1.88–11.30)	0.001	2.44 (0.78–7.64)	0.125
TNM (0 + I + IIvs. III+IV)	6.08 (2.80–13.17)	<0.001	3.80 (1.48–9.76)	0.006	4.84 (1.86–12.60)	0.001	2.53 (0.77–8.26)	0.125
Vessel invasion (yes vs. no)	3.28 (1.56–6.86)	0.002	1.56 (0.58–4.17)	0.376	4.56 (1.84–11.29)	0.001	1.18 (0.38–3.64)	0.772
Perineural invasion (yes vs. no)	1.77 (0.87–3.62)	0.118			1.65 (0.66–4.14)	0.287		
Postoperative complications (yes vs. no)	2.80 (1.37–5.73)	0.005	2.60 (1.15–5.87)	0.022	2.28 (0.93–5.58)	0.071		
GINI	1.01 (1.00–1.01)	0.020	1.00 (1.00–1.01)	0.307	2.97 (1.08–8.20)	0.035	1.01 (1.00–1.02)	0.012

## 4 Discussion

Malnutrition is a prevalent complication during the treatment of malignant tumors, with approximately 80% of cancer patients experiencing varying degrees of malnutrition at different stages of therapy (14). In patients with esophageal cancer, the occurrence of cachexia is particularly prominent. The underlying mechanisms of this condition include reduced food intake and metabolic disorders, which encompass increased energy expenditure, excessive catabolism, and systemic inflammatory responses (15). Moreover, chronic inflammation is recognized as a key characteristic of cancer, influencing the entire process of tumor development and progression (16). In light of recent findings, the importance of nutrition and inflammation-related indicators in assessing prognosis has gained significant attention. Serum albumin levels are not only indicative of the body’s nutritional reserves, but low levels (hypoalbuminemia) are also closely linked to poor postoperative recovery and the onset of cachexia (17). Additionally, C-reactive protein (CRP), a vital marker of systemic inflammation, usually suggests a worse prognosis when present at elevated levels (18); neutrophils contribute to disease progression by promoting tumor cell proliferation, suppressing immune function, and stimulating angiogenesis (19); Conversely, lymphocytes play a crucial role in antitumor immunity through their cytotoxic effects (20). Moreover, platelets enhance tumor angiogenesis and invasive behavior by releasing various bioactive factors (21).

GINI combines key indicators to create a comprehensive biomarker that reflects an individual’s nutritional, inflammatory, and immune status. This index is calculated by multiplying the C-reactive protein/albumin ratio (CAR) by the pan-immunological inflammation value (PIV) (10). Previous studies have shown that CAR is an effective tool for assessing both systemic inflammation and nutritional status (22, 23). As highlighted by Topkan et al. (10), GINI’s primary advantage is its ability to simultaneously incorporate both albumin and CRP—two critical parameters essential for accurate diagnosis. In cancer-associated inflammatory states, inflammatory cytokines such as IL-6 and TNF-α not only stimulate the synthesis of CRP (24, 25) but also inhibit the synthesis of albumin, accelerate its degradation, and promote vascular extravasation (26). GINI effectively captures the fundamental aspects of this pathophysiological process, indicating its potential for predicting treatment efficacy and prognosis. Existing studies confirm that GINI is strongly associated with prognosis across various malignancies. For instance, Bozkurt et al. (12) found that GINI correlated with poor prognosis in metastatic colorectal cancer; Yamamoto et al. (6) identified GINI as an independent predictor of overall survival following radical treatment for esophageal cancer; and Topkan et al. (27) reported that baseline GINI is an effective predictor of progression-free survival and overall survival in patients with glioblastoma.

In recent years, nICT has emerged as a crucial treatment strategy for locally advanced ESCC; however, reliable nutrition-related biomarkers for early prediction of treatment response remain lacking. This study is the first to systematically evaluate the impact of GINI on pathological tumor regression response and prognosis in ESCC patients receiving nICT. The results showed that GINI and lymphocyte count served as independent predictive factors for pathological tumor regression response. The optimal cutoff value of GINI determined by the Youden index effectively stratified patients into subgroups with significant clinical differences. Our analysis demonstrated that patients in the low-GINI group exhibited favorable pathological characteristics, including better tumor differentiation and earlier disease stage, and achieved superior survival outcomes. These findings suggest that GINI serves as a robust biomarker for pretreatment risk stratification. This indicator helps identify high-risk patients who may respond poorly to nICT, providing a basis for developing intensified adjuvant treatment strategies for the high-GINI population, thereby advancing the progress of individualized precision therapy for esophageal squamous cell carcinoma.

Furthermore, in this study, we systematically investigated the association between GINI and clinical outcomes in ESCC patients following nICT using multiple analytical methods. Kaplan–Meier survival analysis demonstrated that patients in the low-GINI group had significantly better survival outcomes. Multivariate Cox regression analysis further confirmed that GINI was an independent prognostic factor for overall survival, reinforcing its biological value in comprehensively reflecting the body’s inflammatory, nutritional, and immune imbalance status. However, it is noteworthy that GINI did not demonstrate independent predictive value for disease-free survival in multivariate analysis. We speculate that this may stem from GINI’s influence on recurrence risk being largely indirect, potentially mediated through intermediate factors such as tumor burden and postoperative complications. In contrast, GINI’s independent predictive power for overall survival suggests that nutritional-inflammatory status may more directly determine long-term prognosis through non-recurrence pathways, including effects on treatment tolerance and non-cancer-related survival. This finding highlights the need in clinical practice to consider not only tumor characteristics but also the host’s systemic response status.

Limitations of this study include: (1) The single-center retrospective design may introduce selection bias; (2) The optimal cutoff value for GINI should be validated in prospective, multicenter observational studies rather than additional retrospective analyses to confirm its generalizability and clinical utility; (3) This study focused on validating the prognostic value of GINI itself. We did not compare its predictive performance with other established or emerging biomarkers in ESCC, such as PD-L1 combined positive score (CPS) or tumor mutational burden (TMB), as their assessment was beyond the scope of this retrospective analysis; (4) Only baseline GINI values were used without considering dynamic changes during treatment; (5) Although GINI demonstrates good comprehensive predictive capability, its specific predictive mechanisms in the context of nICT require further in-depth investigation; (6)The nICT regimen in this study included one of three different PD-1 inhibitors. Due to the limited sample size, we were unable to conduct a thorough subgroup analysis and therefore could not evaluate the effect of each PD-1 inhibitor on treatment efficacy. As a result, we cannot rule out the possibility of unmeasured bias in our analysis arising from the combination of these inhibitors. Future research should focus on establishing standardized cutoff values, implementing dynamic monitoring, elucidating the underlying predictive mechanisms, and exploring whether GINI has a complementary or substitute relationship with other biomarkers.

## 5 Conclusion

This study demonstrates that the pretreatment GINI serves as an effective biomarker for predicting treatment response and prognosis in patients with locally advanced ESCC undergoing nICT. A higher pretreatment GINI level was independently associated with unfavorable pathological characteristics, lower tumor regression rates, and significantly shorter survival. By identifying high-risk patients before nICT, GINI paves the way for more individualized therapy. For instance, patients identified as high-risk by an elevated GINI score could be candidates for more intensive neoadjuvant regimens, closer monitoring during treatment, or be prioritized for subsequent adjuvant therapies. Conversely, low-GINI patients, who are likely to respond well to standard nICT, could be spared from unnecessary treatment intensification and its associated toxicities. The integration of GINI into clinical decision-making, however, requires further validation through multicenter prospective trials.

## Data Availability

The datasets presented in this study can be found in online repositories. The names of the repository/repositories and accession number(s) can be found in the article/Supplementary material.
