# Supplementary material for: The global immune-nutrition-inflammation index predicts pathological response and survival in esophageal squamous cell carcinoma treated with neoadjuvant immunochemotherapy
Source: Front Nutr. 2025 Dec 3;12:1717477. doi: 10.3389/fnut.2025.1717477 (PMC12708532; doi:10.3389/fnut.2025.1717477)
Supplement: Supplementary file 1 [file Table_1.docx]

**Supplemental Table 1.Multivariate Analyses of the GINI Index on Pathological Response and Overall Survival Using Different Scaling Units**

| **Scaling Method** | **Pathological Response (Logistic Regression)** | | **Overall Survival (Cox Regression)** | |
| --- | --- | --- | --- | --- |
|  | **OR (95% CI)** | **P-value** | **HR (95% CI)** | **P-value** |
| **Per 1-unit increase** | 1.05 (1.03-1.06) | <0.001 | 1.01 (1.00-1.02) | 0.012 |
| **Per 1-SD increase (61.30 units)** | 15.252 (6.216-37.422) | <0.001 | 1.909 (1.155-3.156) | 0.012 |
| **Per 25-unit increase** | 3.038 (2.107-4.381) | <0.001 | 1.302 (1.060-1.598) | 0.012 |
| **Per 50-unit increase** | 9.230 (4.439-19.193) | <0.001 | 1.695 (1.125-2.553) | 0.012 |

**Abbreviations: OR, Odds Ratio; HR, Hazard Ratio; CI, Confidence Interval; SD, Standard Deviation.**

**Supplemental Table 2: Sensitivity Analysis Results**

| **Cutoff value** | **sensitivity (%)** | **specificity(%)** | **Positive Predictive Value(PPV, %)** | **Negative predictive value(NPV, %)** |
| --- | --- | --- | --- | --- |
| 72.72 | 0.846 | 0.849 | 77.2 | 90.1 |
| 73.47（最佳） | 0.865 | 0.849 | 77.6 | 91.3 |
| 74.6 | 0.865 | 0.837 | 76.3 | 91.1 |
| 75.415 | 0.885 | 0.826 | 75.4 | 92.2 |
